# Effects of neck pain on reaching overhead and reading: a case–control study of long and short neck flexion

**DOI:** 10.1186/2052-1847-5-21

**Published:** 2013-10-11

**Authors:** Marissa K Constand, Joy C MacDermid

**Affiliations:** 1 School of Rehabilitation Science, McMaster University, 1280 Main Street West, L8S 4 L8, Hamilton, Ontario, Canada

**Keywords:** Neck pain, Reading, Reaching, Long and short neck flexion

## Abstract

**Background:**

Reaching overhead and reading are tasks that many individuals encounter daily. The level of difficulty of these tasks increases if an individual has neck pain. This study determined the neck movement patterns during these two tasks by comparing neck flexion of individuals with and without neck pain.

**Methods:**

This case control study used the portable video technology, Dartfish ProSuite 5.5 Video Software, to analyse neck flexion movement patterns. Healthy individuals and individuals with neck pain were videotaped while they completed two tasks: reaching overhead from a standing position and reading from a sitting position. A single position of interest was selected for analysis from both tasks. The degree of neck flexion presented by the participant in this position at the beginning and end of the task was recorded. The angle change between these two time points was calculated for each participant. Differences between groups were determined by comparing the average flexion angle changes in groups by t-tests.

**Results:**

The average angle change experienced by controls and neck pain participants during the overhead reaching tasks were very similar and a significant difference was not observed. The average angle changes experienced by the two groups during the reading task were more variable, but not significantly different. A t-test comparing average neck flexion angle change during dominant arm elevation for controls (m = −5.28˚, sd = 31.14) and neck pain participants (m = 5.07˚, sd = 32.41) revealed a mean between group difference of −10.34˚ (t_17_ = −0.688, p = 0.5003). The average neck flexion angle change during long neck flexion was not statistically different between controls (m = 10.08˚, sd = 18.89) and neck pain participants (m = 4˚, sd = 18.18); although the mean between group difference was 6.08˚ (t_17_ = 0.6856, p = 0.5022).

**Conclusions:**

Task performance is highly variable between individuals making it difficult to assess the impact of neck pain on small samples even with detailed motion analysis. Despite this, there was a difference in neck posture during reaching activities between controls and patients with neck pain. Neck pain can therefore influence the movement patterns used during daily activities. This has implications for primary and secondary prevention.

## Background

The prevalence of neck pain in the general population is high, as approximately 70% of adults will experience neck pain in their lifetime [1]. It is therefore important to determine how neck pain impacts the performance of daily tasks such as reaching and reading, as these are problematic tasks for people with neck pain. Injury, occupational factors, and psychological factors have all been implicated in neck pain [2]. Previous studies have demonstrated that neck pain causes reduced activation of muscles involved in a repetitive upper limb task; potentially as a mechanism to mitigate a painful stimulus [3]. Such reduced muscle activation could result in either poor coordination of movement or reduced arcs of motion. Neck pain has also been found to lead to muscle fatigue, which can negatively impact an individual’s posture, muscle velocity, muscle power output, and ability to complete repetitive movements [3,4]. Self-report scales are sometimes used to collect information on tasks that are difficult with neck pain and can tell us the extent to which different tasks are problematic. However, self-report cannot inform our understanding of the kinematics or anatomy of how tasks are performed differently with neck pain.

It is hypothesized that individuals with neck pain will display reduced arcs of motion, or “bracing”, during reaching and reading tasks when compared to those without neck pain. The assumption associated with this hypothesis is that a correlation exists between neck pain and long and short neck flexion angles. Reaching overhead and reading are functional tasks that require the neck to perform repetitive movements and hold the head in a static position. This study selected two tasks that incorporate different types of cervical motion, with short neck flexion (reach overhead task) representing upper cervical spine motion and long neck flexion (reading task) representing lower cervical spine motion. Due to the fact that the two primary causes of neck pain are repetitive movements and static postures [5], these two tasks were selected to represent different types of challenges that might be compromised in people with neck pain. The purpose of this study was to determine whether differences exist in the arc of motion for people with or without neck pain.

## Methods

Twelve healthy subjects and seven subjects with neck pain were recruited from November 2011 to April 2012. All subjects were over 18 years of age and gave informed written consent for their participation in the study. Inclusion criteria for neck pain participants included the reporting of neck pain on the Neck Disability Index. Exclusion criteria for both subject groups included any underlying neurological condition and consumption of alcohol or caffeine four hours prior to their lab appointment. Due to the small sample size, the study was not powered for subgroup analysis. Ethics approval for this project was granted by the Hamilton Health Sciences Research Ethics Board.

**Figure 1:**
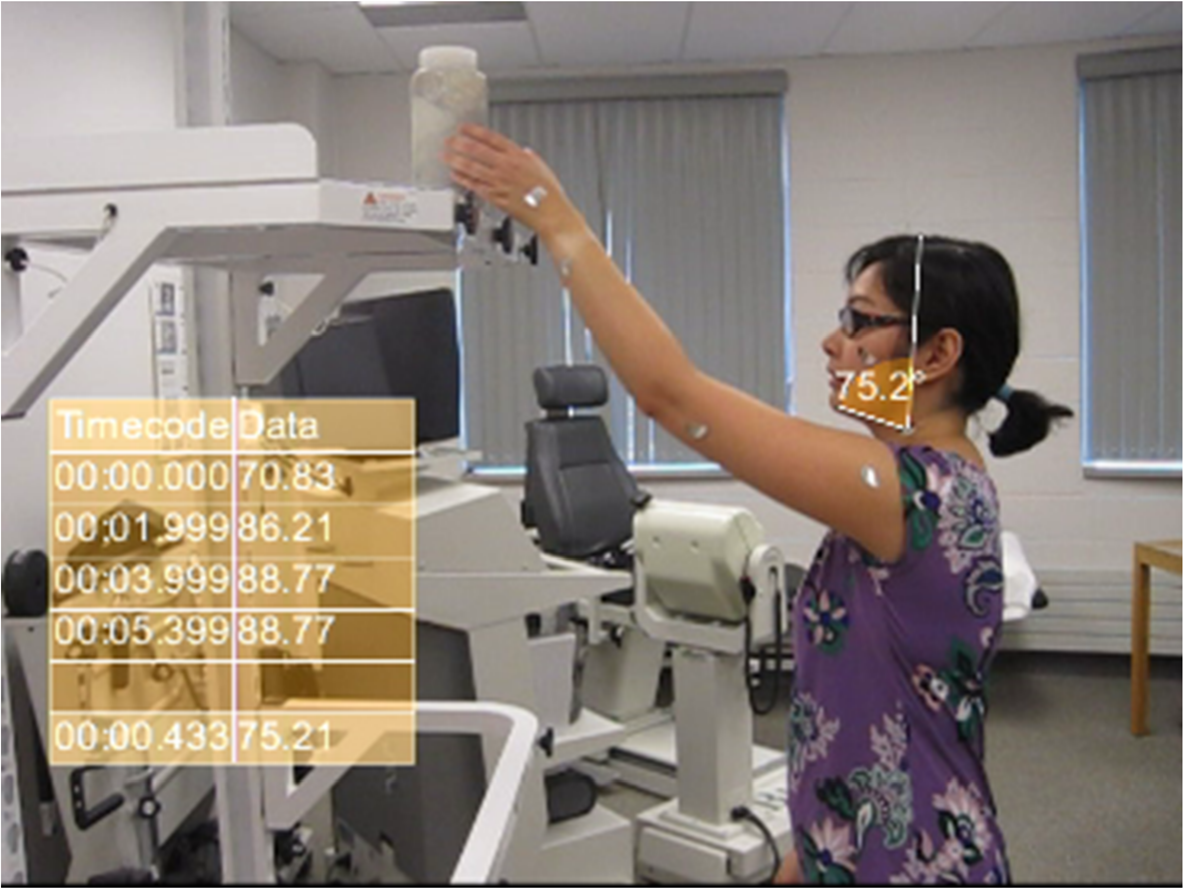
**Participant during overhead reaching task.** This image represents Position A and was taken during the analysis of the neck flexion of the participant reaching overhead.

The two groups compared were controls (n = 12) and participants with neck pain (n = 7). Participants were video recorded using the Canon PowerShot SD1200 IS Digital ELPH camera while performing a standardized reaching task where they were required to reaching overhead to place a two pound weight on a shelf from a standing position using their dominant arm [6,7]. The shelf was placed 25 inches above the participant’s naval. This action was repeated for 30 seconds to a metronomic beat set at 30 beats per minute indicating to the participant when to elevate and lower the weight from the shelf. Participant movement during the overhead reach was recorded in the sagittal plane. Short neck flexion was tracked in Position A by consistently tracking and measuring the angle between the chin and the plumb line for 30 seconds (Figure 1).

**Figure 2:**
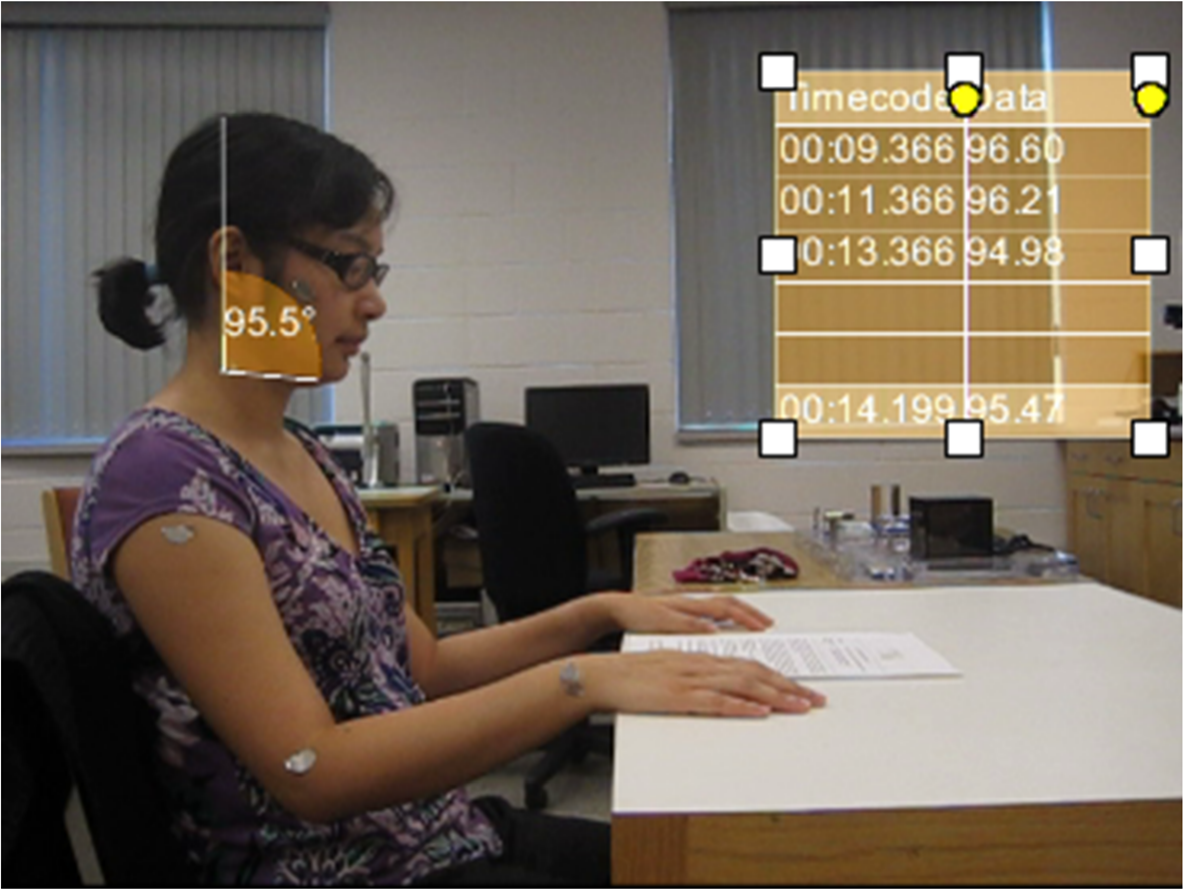
**Participant during reading task.** This image represents Position B and was taken during the analysis of the neck flexion of the participant reading.

Participants were then recorded while reading a text in a sitting position for two minutes. Data capture was extended to two minutes for the reading task, as it is identified by Murphy, Buckle & Stubbs [8] that long neck flexion positions will vary with time. The text to be read was resting flat on a table top. Participant movement during the reading task was recorded in the sagittal plane. Long neck flexion was tracked in Position B by measuring the angle between the chin and the plumb line for one minute (Figure 2).

Dartfish ProSuite 5.5 Video Software Solutions was used to analyse movement in two dimensions. This two dimensional analysis is consistent with the definitions of short and long neck flexion employed in this study. Using the angle tracking tool, neck flexion was measured and tracked using the Dartfish Analyzer Data Table, which recorded angle measurement and its associated time code. Position A’s neck flexion angles were measured at baseline (time 0) and at task completion (30 seconds post task initiation). Position B’s neck flexion angles were also measured at baseline (time 0) and at task completion (30 seconds post task initiation). The angles at task completion were subtracted from those at baseline to determine the number of degrees by which the angle changed (Tables 1, 2, 3 and 4). For the purpose of this study, this calculation represented the arc of motion experienced by the participants for each task being analysed, and served as the data upon which the two groups were compared. All statistical values were determined by Stata IC12 Statistical Software. All between group analyses of neck flexion angle changes were conducted using a t-test.

**Table 1:** Overhead reaching task angle changes in controls

	**Initial**	**Final**	**Angle change**	**Mean angle**
	**angle (˚)**	**angle (˚)**	**difference (˚)**	**change (˚)**
1	71.39	54.76	16.63	−5.28
2	88.75	132.65	−43.9	
3	48.18	122.94	−74.76	
4	87.28	96.88	−9.6	
5	92.62	43.83	48.79	
6	94.53	88.67	5.86	
7	90.42	79.76	10.66	
8	101.72	91.73	9.99	
9	90	107.3	−17.3	
10	90.84	83.95	6.89	
11	81.17	90.8	−9.63	
12	93.39	100.42	−7.03	

**Table 2:** Overhead reaching task angle changes in neck pain participants

	**Initial**	**Final**	**Angle change**	**Mean angle**
	**angle (˚)**	**angle (˚)**	**difference(˚)**	**change (˚)**
1	92.82	99.26	−6.44	5.06
2	92.57	44.45	48.12	
3	66.31	70.88	−4.57	
4	95.07	150.42	−55.35	
5	104.4	90.6	13.8	
6	112.6	87.5	25.1	
7	101.3	86.5	14.8	

**Table 3:** Reading task angle changes in controls

	**Initial**	**Final**	**Angle change**	**Mean angle**
	**angle (˚)**	**angle (˚)**	**difference(˚)**	**change (˚)**
1	94.78	88.52	6.36	10.08
2	116.49	66.46	50.03	
3	85.96	69.31	16.65	
4	94.63	97.24	−2.61	
5	94.53	88.67	5.86	
6	145.34	106.85	38.49	
7	123.63	123.62	0.01	
8	102.65	92.1	10.55	
9	112.18	105.35	6.83	
10	112.94	104.6	8.34	
11	108.2	104.1	4.1	
12	109.96	133.6	−23.64	

**Table 4:** Reading task angle changes in neck pain participants

	**Initial**	**Final**	**Angle change**	**Mean angle**
	**angle (˚)**	**angle (˚)**	**difference (˚)**	**change (˚)**
1	89.28	81.25	8.03	4.00
2	116.7	133.19	−16.49	
3	143.68	141.56	2.12	
4	101.51	61.67	39.84	
5	127.8	138.5	−10.7	
6	130.1	123	7.1	
7	104	105.9	−1.9	

## Results

Changes between baseline, or resting position, and final neck flexion angles were represented by positive numbers if the final angle was smaller than the initial angle, and negative numbers if the final angle was greater than the initial angle (Tables 1, 2, 3 and 4).

A t-test was used to compare average neck flexion angle change during dominant arm elevation for controls (m = −5.28˚, sd = 31.14) and neck pain participants (m = 5.07˚, sd = 32.41) to determine if there were differences in arcs of motion used during an overhead reaching task. Non-significant differences in arcs of motion were observed between controls and patients with neck pain, as a t-test revealed a mean between group difference of −10.34 (t_17_ = −0.688, p = 0.5003).

A t-test comparing average neck flexion angle change during the reading task for controls (m = 10.08˚, sd = 18.89) and neck pain participants (m = 4˚, sd = 18.18) was conducted to determine differences in arcs of motion during a reading task. A mean between group difference of 6.08 was observed, representing a non-statistically significant difference (t_17_ = 0.6856, p = 0.5022).

It was observed that participants completed the reaching task in two main ways. They either maintained their gaze in the forward direction while they raised and lowered the weight, or they followed the weight with their eyes. Those that chose the second strategy were observed to have a greater variance in their neck flexion. In particular, it was observed that the older participants were more likely to use this strategy.

## Discussion

This study provided preliminary results demonstrating small differences in task performance between people with neck pain and those without neck pain performing standardized tasks; and the feasibility of assessing differences in task performance using two-dimensional video-based movement analysis. Although the current gold standard for kinematic analysis is three dimensional tracking of angles, Dartfish Video Software Solutions, a two dimensional video-based tracking system, has potential advantages for clinical assessment because it allows assessment of task performance in different contexts, is much more economical than a movement laboratory, and provides an option to review performance over time for retraining purposes. Others have suggested the usefulness of this approach for assessing lower limb movement [8-11]; however, this study adds that we were able to successfully monitor changes in neck flexion. This is important since neck motion is quite different than measurements that would be taken for the lower limb. Borel, Schneider, & Newman [10] identify that the software’s “ease of use” allows for studies to track movement for longer periods of time, as the software requires less time to complete tasks than other visual assessments of kinematics. This is particularly useful for studying problems aggravated by repetitive movements over extended periods of time, such as those that cause neck pain. Miller & Callister [11] identify that using the software to track movement results in high intra-rater reliability because even those new to Dartfish Video Software Solutions were able to accurately follow software instructions to create data.

Functional tasks are complex movements that are influenced by motor control, body size and shape, and many other factors. A variety of motor control theories exist to describe how movement takes place. The theory of abundance states that a limb has many degrees of freedom [12], and implies that there are many different strategies that can be used to accomplish a specific motor goal. In this study, we provided a standardized task that allowed some flexibility in how it was performed. However, both anthropometric factors and motor control factors undoubtedly contributed to the wide variation we saw between individuals and how these tasks were performed. When reaching overhead, individuals have an abundant number of potential strategies to lift their arms to reach and grasp the weight from the shelf. Every time the participant reached for the weight during the overhead reach task, the central nervous system employed its muscles and joints differently [12]. Thus, there is some variation even within a single individual on how tasks are repeatedly performed.

People with neck pain may need to alter their strategies in performing tasks to accommodate their neck pain. Changes in strategies could influence the speed of motion, the path of motion taken, the arc of motion used, the coordination of the motion and other parameters. In this study, we focused on measuring the arc of motion used to perform the task. Thus, changes in motor strategies in other parameters such as speed of motion were not reflected in our analysis and may have been missed. Our study may not have captured different forms of compensation use by patients to minimize neck pain such as relying more heavily on upper limbs and postural muscles to achieve the reaching task.

Unfortunately, the variant of eye gaze previously identified in the results was not anticipated and was only noted once data collection began; so formal measurement of eye gaze was not performed in all subjects. A potential reason for older individuals to require more visual feedback to maintain their grip on an object is that they may have diminished sensory feedback about the nature of their grip [13]; they may have a lower grip strength and be gripping with a different operational range that younger individuals; or, they may have less confidence in their ability to maintain the activity and require visual feedback to increase their focus on the task [14].

Although motion analysis is often completed with small sample sizes, variability in task performance was greater than we had anticipated. Another potential source of variation in how people perform tasks is based on their anthropometric characteristics. Although the height of the reaching task was related to the person’s eye level, and hence had some compensation for height; it is likely that tasks vary by body size and gender. Future studies should consider matching on these factors to reduce this variation. The overall effect of variability between subjects whether due to anthropometric reasons, motor control reasons, or others contributed to variation between subjects beyond our initial estimates. The effect was reduced power in our statistical analyses. Hence, group differences of 10° in neck flexion which might be considered clinically significant by some, were not statistically significant.

There are also limitations in our study in relation to the use of video analysis. As Norris & Olson [9] have identified, a limitation to using Dartfish Video Software Solutions is that the number of studies tracking movements of participants with varying pathologies published today is small, and hence there is a lack of reference values or parameters for subject variability/error comparisons purposes. Although the software was able to track neck flexion in this study, a comparison with the gold standard of kinematics tracking would be useful to assess how accurately Dartfish Video Software Solutions was able to track differences in neck flexion over time. Obviously, two-dimensional analysis provides less information than three dimensional analysis. In this study, we restricted ourselves to a two-dimensional analysis because we are interested in neck flexion/extension arc of motion. Video analysis can include three-dimensional considerations when two cameras are used, as long as these are synced. Spinal motion is complex, and video analysis is not the optimal approach to investigate at what level motion was occurring. We know that flexion occurs differently at different levels of the spine. More specifically, upper cervical spine motion includes movement of the atlas (C1) and the odontoid process (C2) [15], and this study has referred to flexion at this C1-C2 craniocervical junction as short neck flexion. Furthermore, long neck flexion is associated with flexion at the lower cervical spine via the separation of spinous processes of C2-C7 vertebrae [15]. A final limitation to this study is the fact that trunk movement was not restricted during the trials. However, it should be noted that excessive restriction placed on subjects could have led to unnatural task completion.

Left untreated, acute neck pain can become chronic and result in secondary health problems [4]. For this reason, research into the causes and effects of neck pain is imperative, especially in today’s aging society where 90% of office workers use computers daily (computer work being cited as contributing to neck pain and postural muscle fatigue) [16]. Gender, age, poor social support, job dissatisfaction, and high job demands all contribute to the development of neck pain [17]. Driessen et al. [17] identify that research into prevention is “scarce”, and due to the “multifactorial origin” of neck pain, it is necessary to educate individuals on how to prevent neck pain. Although our study is preliminary, it does provide preliminary information on the amount of neck motion used, and the types of motor strategies employed during two standardized functional tasks. Understanding how functional and work tasks are performed, and how this might contribute to neck disorders is an important and understudied area. Larger studies of task performance in different contexts such as workplaces, and in larger groups of individuals considering personal and environmental factors are needed to fully understand the exposures which might be contributing to neck pain.

## Conclusions

This study found that video analysis was able to measure neck flexion, arcs of motion, and movement strategies that were highly variable between individuals when performing two standardized optional tasks involving upper and lower cervical spine flexion. Although mean differences in arcs of motion during reading were 10° different for people with neck pain in comparison to controls, larger sample sizes will be needed to obtain statistical significance. It is therefore important to note that armed with the knowledge on how to minimize risk factors for neck pain, individuals will hopefully be able to modify their habits in order to maintain their musculoskeletal health and thus ameliorate their quality of life.

## Consent

The individual pictured in the figures associated with this manuscript has provided written consent for inclusion of these images.

## Competing interests

The authors identify no competing interests.

## Authors’ contributions

This study was completed as part of the undergraduate thesis work of MKC who was the principal investigator in this study. MKC and JCM collaborated to create a study design to answer the research questions posed. MKC recruited participants, collected data, and analyzed data. JCM reviewed the data collected and provided essential guidance and direction for the data analysis. Both authors read and approved the final manuscript.

## Pre-publication history

The pre-publication history for this paper can be accessed here:

http://www.biomedcentral.com/2052-1847/5/21/prepub
